# Transarterial Embolization for Refractory Non-Cervical-Origin Interscapular Pain Following Ultrasound-Guided Injection: A Retrospective Feasibility Study

**DOI:** 10.3390/diagnostics15192496

**Published:** 2025-10-01

**Authors:** Yu-Han Huang, Chia-Wei Chang, Jui-Yuan Chen, Chia-Shiang Lin, Chun-Wei Lin, Ping-Sheng Lu, Neng-Yu Chiu, Keng-Wei Liang

**Affiliations:** 1 Interventional Pain Management Center, Department of Anesthesiology, Shin Kong Wu Ho-Su Memorial Hospital, Taipei 111045, Taiwan; joyce332@gmail.com (Y.-H.H.); ryuanglavine@gmail.com (J.-Y.C.); waynehorn109@gmail.com (C.-W.L.); 2 Department of Orthopedic Surgery, Chang Gung Memorial Hospital, Keelung Branch, Keelung 20401, Taiwan; flyinwei@gmail.com; 3 School of Medicine, Fu-Jen Catholic University, New Taipei City 24205, Taiwan; 4 Department of Anesthesiology, Shuang Ho Hospital, Taipei Medical University, New Taipei City 235603, Taiwan; seancslin@gmail.com; 5 Department of Anesthesiology, School of Medicine, College of Medicine, Taipei Medical University, Taipei 11031, Taiwan; 6 College of Medicine, Department of Radiology, Kaohsiung Medical University, Kaohsiung 807378, Taiwan; 7 Department of Medical Imaging, Landseed International Hospital, Taoyuan 324609, Taiwan; felysnoopy@gmail.com; 8 Department of Medical Imaging, Chung Shan Medical University Hospital, Taichung 402306, Taiwan; noelchiu2009@gmail.com; 9 School of Medicine, Chung Shan Medical University, Taichung 402306, Taiwan

**Keywords:** transarterial embolization, interscapular pain, ultrasound-guided injection, chronic musculoskeletal pain, imipenem/cilastatin sodium

## Abstract

**Objective**: Chronic non-cervical-origin interscapular pain remains challenging to treat when refractory to conservative management and ultrasound-guided injections. This retrospective feasibility study aimed to assess the feasibility, procedural practicality, safety, and preliminary clinical outcomes of transarterial embolization (TAE) as a salvage therapy in this patient population. **Methods**: This single-center retrospective study included 20 patients with chronic interscapular pain (Numeric Rating Scale [NRS] score ≥5 for >3 months) who initially underwent ultrasound-guided injection therapy. Patients who experienced inadequate pain relief after 3 months (*n* = 10) proceeded to TAE, while the remaining 10 patients with sufficient relief formed the comparison group. TAE primarily targeted the transverse cervical artery using imipenem/cilastatin sodium as the embolic agent. Pain outcomes were assessed using NRS scores at 1, 3, and 6 months post-procedure. The primary outcome was pain reduction (≥50% decrease in NRS score), with secondary outcomes including technical success, medication use, and safety assessment. **Results**: The mean baseline NRS score for all patients was 6.5 ± 1.4, which decreased to 3.4 ± 2.0 at 1 month and 3.9 ± 2.5 at 3 months post-injection (*p* < 0.001). In the TAE group, the NRS score decreased from 7.4 ± 1.4 to 5.1 ± 1.1 at 1 month and 6.0 ± 1.4 at 3 months, indicating inadequate pain relief. In contrast, the injection-only group showed significant improvement, with NRS scores decreasing from 5.6 ± 0.5 to 1.6 ± 0.5 at 1 month and 1.7 ± 0.7 at 3 months (*p* < 0.001). The reduction in NRS scores was significantly less in the TAE group compared with the injection-only group (−2.2 vs. −4.0 and −28.7% vs. −71.4% at 1 month; −1.4 vs. −3.9 and −18.2% vs. −69.7% at 3 months; all *p* ≤ 0.001). Following TAE, the mean NRS score further decreased to 2.1 ± 0.7, 2.0 ± 1.1, and 1.9 ± 1.2 at 1, 3, and 6 months, respectively (*p* < 0.001), with clinical success rates of 90%, 100%, and 90% at these respective time points. At the final follow-up, the percentage of NRS score reduction was comparable between the TAE and injection-only groups (−74.8% vs. −69.7%, *p* = 0.397). No severe or life-threatening adverse events were observed; only self-limited adverse events were reported. **Conclusions**: In this retrospective feasibility study, TAE appeared safe and effective as a salvage therapy for patients with refractory non-cervical-origin interscapular pain unresponsive to injection therapy. Further prospective, randomized studies are needed to validate these findings, refine patient selection criteria, and optimize treatment outcomes.

## 1. Introduction

Chronic interscapular pain, typically localized to the medial scapular region around the T3–T8 thoracic levels, represents a distinct form of upper back pain. Lifetime prevalence estimates suggest that 15% to 19% of individuals experience chronic upper back pain [1]. In severe cases, chronic interscapular pain can significantly disrupt sleep, mood, work productivity, concentration, and overall quality of life [2].

The etiology of chronic interscapular pain is complex and multifactorial. Commonly, it arises as referred pain from lower cervical nerve roots, facet joints, or intervertebral discs, often manifesting as discomfort in the lower neck or interscapular region [3]. Thoracic facet joints and costotransverse joints may also contribute to similar symptoms [4,5]. Additionally, excessive strain on upper back muscles—frequently associated with prolonged desk work or poor posture—can result in pain originating from muscle tension or nerve entrapment within the intermuscular fascia [6,7,8]. Due to its diverse causes, chronic interscapular pain often requires a multimodal treatment approach tailored to the underlying etiology. Conservative treatments, including oral medications, posture correction, and rehabilitation exercises, typically serve as the first-line therapy. If these initial measures fail to provide satisfactory relief, invasive interventions may be considered. Pain originating from the cervical spine may require targeted interventions, while thoracic facet joint-related pain is effectively managed with procedures like medial branch blocks and thoracic facet joint injections. A study by Lee et al. reported that thoracic medial branch blocks and direct intra-articular facet joint injections provided sustained pain relief in 40% and 65% of patients, respectively, after six months [9]. A study by Modi et al. reported that 40% of patients achieved a minimal clinically important difference in interscapular mid-thoracic myofascial pain at 12 weeks after undergoing combined ultrasound-guided trigger point injections and nerve hydrodissection [10]. Despite these advances, a subset of patients remains refractory to current therapies. This underscores the need for innovative and more effective treatment strategies to address chronic interscapular pain.

Transarterial embolization (TAE) was first introduced in 2013 as a treatment for chronic tendinopathy and enthesopathy and has since been applied to various musculoskeletal conditions with promising results [11,12,13]. By inhibiting abnormal angiogenesis, TAE has demonstrated effectiveness in reducing pain and inflammation in both animal models and human studies of frozen shoulder [14,15]. Trapezius myalgia, considered a type of interscapular myofascial pain, was first treated with TAE by Shibuya et al., primarily targeting the transverse cervical artery [16]. Their study reported an average pain reduction of more than 50% and a clinical success rate of 71.4% at six months; however, only 17% of patients in that cohort had received trigger-point injections before TAE. Given the limited research on the efficacy of TAE for chronic interscapular pain in patients with suboptimal responses to ultrasound-guided injection therapy, further investigation is warranted. Accordingly, this work was designed as a retrospective feasibility study to assess procedural practicality, short-term safety, and the signal of clinical efficacy of TAE in patients with interscapular pain refractory to ultrasound-guided injections. The feasibility framing was pre-specified to guide sample size planning and endpoint selection for subsequent prospective trials.

## 2. Materials and Methods

### 2.1. Study Design and Ethics

This was a single-center retrospective feasibility study approved by the institutional review board; data were obtained from electronic medical records. Informed consent was obtained from patients for the TAE procedure.

### 2.2. Treatment Algorithm and Patient Selection

As part of this feasibility design, a tailored, stepwise management approach to interscapular pain was employed. Patients with suspected cervical-origin pain were excluded, as their management differs. Cervical origin was suspected in patients who presented with radicular symptoms in the upper extremities, cervical facet tenderness, or neurological deficits on physical examination. When clinically indicated, cervical spine MRI or radiographs were performed, and patients demonstrating findings such as disc herniation, foraminal stenosis, or facet arthropathy consistent with a cervical etiology were excluded from this study.

All included patients initially underwent conservative treatment, which comprised oral medications and rehabilitation. Those who did not experience adequate pain relief were then considered for ultrasound-guided injection therapy. The injection regimen and location were determined based on the clinician’s assessment and discussion with the patient, and included options such as intramuscular steroid injections, intramuscular high-concentration dextrose injections, intramuscular platelet-rich plasma injections, dorsal scapular nerve blocks/hydrodissection, and medial branch blocks of the thoracic spine. Following the injection, patients were scheduled for follow-up appointments at 1 month and 3 months to assess pain response. Patients with inadequate pain relief after these follow-up assessments underwent TAE. All patients who underwent TAE were scheduled for an additional 6-month follow-up.

### 2.3. Patient Population

**Figure 1:**
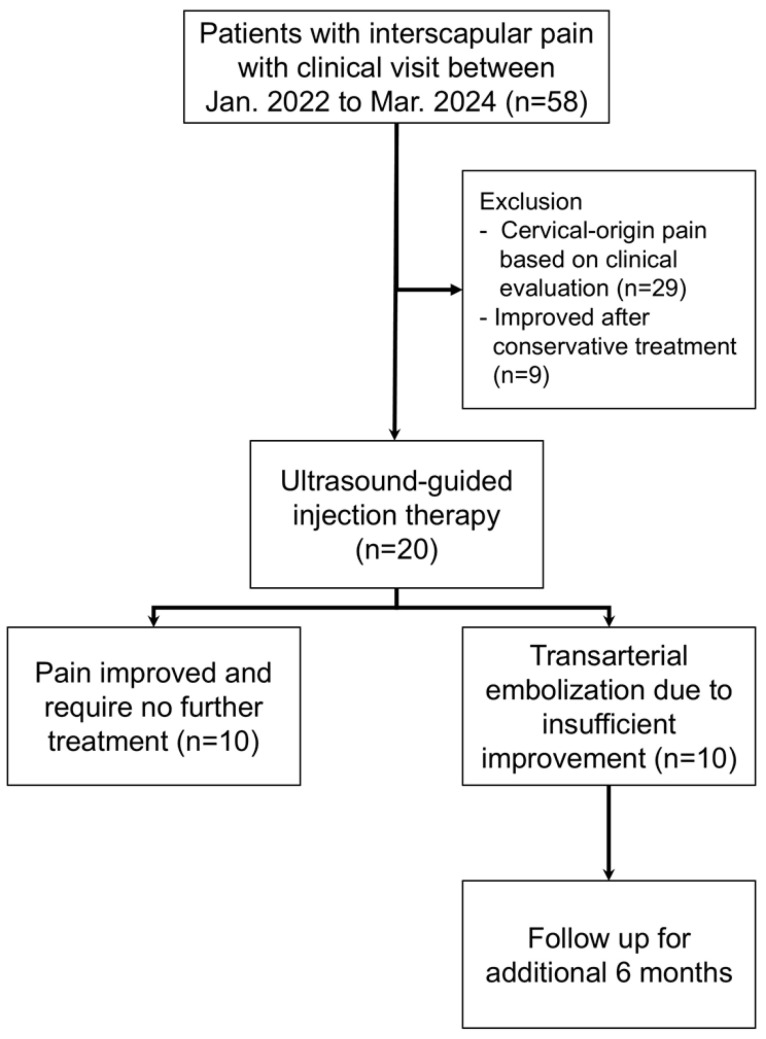
Flow diagram of patient selection, enrollment, and follow-up.

Between January 2022 and March 2024, 58 consecutive patients with moderate-to-severe interscapular pain [Numeric Rating Scale (NRS) ≥ 5] persisting for more than 3 months presented to our clinic. Of these, 29 patients were excluded due to suspected cervical-origin pain based on clinical and imaging evaluation and were directed to alternative treatments. Among the remaining 29 patients, nine achieved satisfactory pain relief with conservative management. The other 20 patients proceeded to ultrasound-guided injection therapy. After 3 months of follow-up, 10 patients showed inadequate pain relief and subsequently underwent TAE, whereas the other 10 experienced substantial and sustained pain relief without further intervention. Thus, 10 patients treated with TAE and 10 patients who responded to injection therapy were included in the final analysis. Consistent with a feasibility study, the target sample reflected consecutive availability and procedural practicality rather than formal power calculations. The treatment algorithm and patient selection process are summarized in Figure 1.

### 2.4. TAE Procedure

All TAE procedures were performed by a single experienced interventional radiologist (Y.H.H.), who has over 12 years of expertise. Under local anesthesia, percutaneous access to the ipsilateral radial artery was obtained using a 5-F introducer sheath. Subclavian digital subtraction angiography (DSA) was performed using a 4-F diagnostic catheter (JR4, Terumo, Tokyo, Japan) with a 15 mL injection of iodinated contrast medium (Xenetix 350, Guerbet, Villepinte, France) at an injection rate of 3 mL/s. The transverse cervical artery was identified via DSA, and superselective catheterization was achieved using either a 4-F or 5-F catheter (JR4, Terumo, Tokyo, Japan, or RIM, Cook Medical, Bloomington, IN, USA) to engage the arterial orifice. A 1.98-F microcatheter (Masters Parkway Soft, Asahi Intecc, Aichi, Japan) was then advanced distally to a segment adjacent to the patient’s painful area. Angiography was performed with a slow, manual injection of 1–2 mL of contrast agent. If abnormal staining was observed or if the patient reported evoked pain—defined as reproducible interscapular pain or a heat sensation corresponding to their usual symptoms during selective angiography—embolization was initiated. The deep branch of the transverse cervical artery, which primarily supplies the interscapular region, was the main target of this procedure. If the patient reported a broader pain distribution extending to the upper trapezius region or the lateral aspect of the scapula, the superficial branch of the transverse cervical artery and the circumflex scapular artery were catheterized and embolized accordingly.

Embolization was performed using a mixture of imipenem/cilastatin sodium (IPM/CS, 500 mg suspended in 10 mL of iodinated contrast), delivered in 0.5–1 mL increments until transient stasis of antegrade blood flow was achieved for 3–5 heartbeats in the target vessel. Typically, 2–5 mL of the IPM/CS mixture were required to reach the treatment endpoint. Following the procedure, the catheter and introducer sheath were removed, and hemostasis was achieved via manual compression or a radial compression device (TR Band, Terumo, Tokyo, Japan).

Patients were monitored for two hours post-procedure before being discharged. If residual or recurrent pain was reported during follow-up, an additional TAE session was permitted at the clinician’s discretion one month after the initial procedure, with the date of the additional treatment recorded in the medical records. Patients were allowed to continue their pre-existing conservative treatment regimens but were restricted from initiating any new interventions for six months post-TAE.

### 2.5. Clinical Outcome Measurements and Safety

Pain severity was assessed using the NRS score at baseline (before any invasive procedure), and at 1 and 3 months after ultrasound-guided injection. For patients undergoing TAE, NRS scores were also recorded at 1, 3, and 6 months post-embolization. Changes in the NRS score were evaluated on the treated side. For patients who received bilateral treatment, pain score changes were assessed on a per-patient basis. Technical success of TAE was defined as successful selective angiography of the transverse cervical artery, effective delivery of the IPM/CS mixture, and achievement of the treatment endpoint (transient stasis of antegrade blood flow). Clinical success was defined as at least a 50% reduction in NRS pain score at each follow-up time point compared to baseline. Baseline was defined as the pre-injection NRS for injection analyses and the pre-embolization NRS for post-TAE analyses. The use of oral medication or prior conservative treatment was documented for patients undergoing TAE at each follow-up, with usage categorized as either “daily use” or “as needed.” Adverse events were recorded and classified according to the guidelines established by the Society of Interventional Radiology [17].

### 2.6. Statistical Analysis

Demographic data were compared between the two groups using an independent *t*-test for continuous variables and the Fisher exact test for categorical variables. Baseline and follow-up NRS scores were analyzed within each group using repeated measures analysis of variance with Bonferroni post hoc testing to assess within-group differences over time. Clinical success rates were calculated as percentages at each follow-up time point. Changes in NRS scores and percentage reductions were determined by subtracting the baseline NRS scores, and comparisons between the two groups were conducted using an independent *t*-test. In the TAE group, changes in NRS scores and percentage reductions at each follow-up time point were compared with the corresponding data from the injection-only group at the 3-month follow-up. All statistical analyses were conducted using SPSS, version 25 (IBM, Armonk, NY, USA) and Prism, version 9 (GraphPad Software, La Jolla, CA, USA). A two-sided *p*-value < 0.05 was considered statistically significant.

## 3. Results

Within the scope of this study, a total of 20 patients, including 9 males and 11 females with chronic interscapular pain, underwent ultrasound-guided injection therapy. The mean age of the patients was 49.1 ± 9.4 years, with a mean pain duration of 31.4 ± 56.6 months. Among them, 10 patients underwent further TAE due to unsatisfactory pain relief, while the remaining 10 patients experienced adequate pain relief and required no additional treatment. The group requiring further TAE treatment had a significantly higher baseline NRS score compared to the group that underwent injection-only therapy (7.4 vs. 5.6, *p* = 0.003), while no significant differences were observed in age, gender, or pain duration between the groups. The demographic data of the patients are presented in Table 1.

### 3.1. Pain Changes After Ultrasound-Guided Injection

Among the 20 patients, the mean baseline NRS score was 6.5 ± 1.4, which significantly decreased to 3.4 ± 2.0 at 1 month and 3.9 ± 2.5 at 3 months after ultrasound-guided injection (overall *p* < 0.001; *p* = 0.003 and *p* = 0.005 at 1 month and 3 months, respectively, when pairwise comparisons were made with baseline). The overall clinical success rate was 55% at 1 month and 50% at 3 months. In patients who underwent subsequent TAE, the baseline NRS score was 7.4 ± 1.4, which decreased to 5.1 ± 1.1 at 1 month and 6.0 ± 1.4 at 3 months. In contrast, the baseline NRS score in patients who received injection-only treatment was 5.6 ± 0.5, decreasing to 1.6 ± 0.5 at 1 month and 1.7 ± 0.7 at 3 months. The reduction in NRS scores and percentage improvement was significantly less in the TAE group compared to the injection-only group (−2.2 vs. −4.0, *p* = 0.001 and −28.7% vs. −71.4%, *p* < 0.001 at 1 month; −1.4 vs. −3.9, −18.2% vs. −69.7% at 3 months, all *p* < 0.001). The clinical success rate was 10% (1/10) at 1 month and 0% (0/10) at 3 months in the TAE group, compared to 100% (10/10) at both 1 month and 3 months in the injection-only group.

### 3.2. Pain Changes After TAE

**Figure 2:**
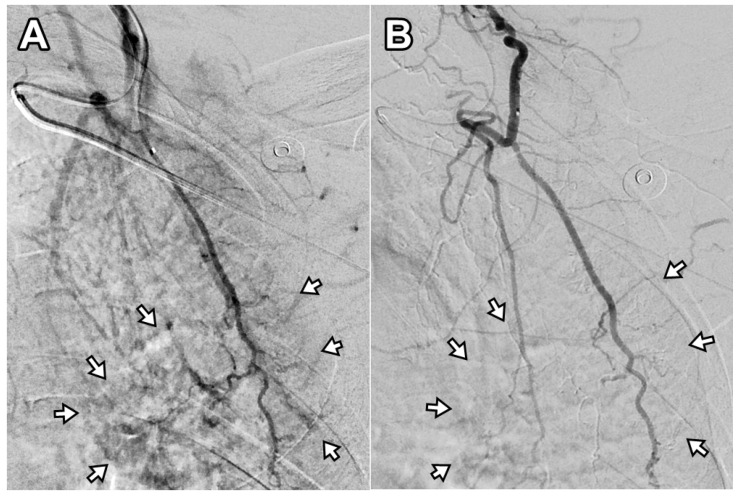
Digital subtraction angiography of the deep branch of the left transverse cervical artery in a 56-year-old woman with refractory interscapular pain. (**A**) Pre-embolization image shows abnormal neovascularization in the interscapular area (arrows). (**B**) Post-embolization image demonstrates disappearance of abnormal blush in the corresponding area (arrows). This patient experienced a decrease in NRS score from 6 to 2 at 6-month follow-up (67% reduction).

**Figure 3:**
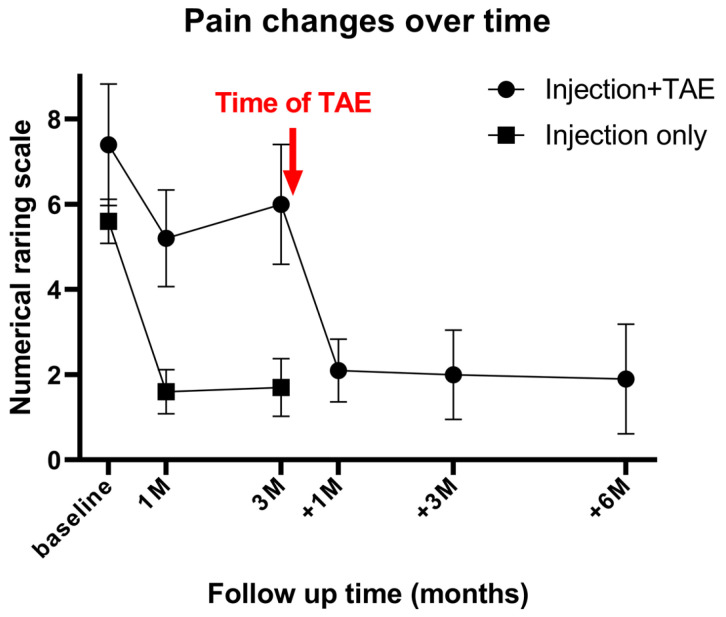
Mean numerical rating scale (NRS) scores over time in patients receiving ultrasound-guided injection only (*n* = 10) and in patients who underwent injection followed by subsequent transarterial embolization (TAE) (*n* = 10). The red arrow indicates the timing of TAE in the latter group. Baseline, 1M, and 3M denote 1 and 3 months after injection; +1M, +3M, and +6M denote 1, 3, and 6 months after TAE. M = month. Patients with inadequate response to injection therapy demonstrated significant pain reduction after TAE, which was sustained for up to 6 months.

Two patients required a second TAE procedure due to persistent pain at 75 and 41 days after the initial treatment. A total of 12 TAE procedures were performed in 10 patients, achieving a technical success rate of 100% (12/12). On average, 1.8 ± 0.9 arterial branches were embolized per procedure, and 171 ± 54.2 mg of IPM/CS was used per TAE. An illustrative case is presented in Figure 2. Following TAE, the NRS score significantly decreased to 2.1 ± 0.7 at 1 month, 2.0 ± 1.1 at 3 months, and 1.9 ± 1.2 at 6 months (*p* < 0.001). Clinical success, defined as an NRS reduction of ≥50% from baseline, was achieved in 90% (9/10), 100% (10/10), and 90% (9/10) of patients at 1, 3, and 6 months, respectively. The reduction in NRS score after TAE was significantly greater at 1 month, 3 months, and 6 months when compared with the last follow-up data of the injection-only group (*p* = 0.024, 0.005, and 0.006, respectively). The percentage of NRS score change was −70.2%, −73.4%, and −74.8% at 1, 3, and 6 months post-TAE, showing no significant difference compared with the last follow-up data of the injection-only group (−69.7%; *p* = 0.931, 0.486, and 0.397 at 1, 3, and 6 months post-TAE, respectively). The changes in NRS score over time are illustrated in Figure 3. A detailed comparison of pain score reductions and percentage changes between groups following injection and TAE is shown in Table 2. Detailed individual patient-level data, including baseline NRS, pain duration, type of injection, TAE details, and follow-up NRS, are provided in Table S1.

### 3.3. Frequencies in Conservative Treatment Use and Safety Profile

Among the 10 patients who required TAE treatment, 100% used oral NSAIDs, 70% used oral opioids, and 100% participated in daily rehabilitation prior to TAE. A decreasing trend was observed in the use of these treatments, both daily and as needed, at 1, 3, and 6 months post-TAE. The trend in decreased medication use is summarized in Table 3.

No severe or life-threatening adverse events were reported. Mild, self-limited adverse events included puncture site hematoma (*n* = 1; 8.3%), cutaneous erythema (*n* = 2; 16.7%), transient radial artery spasm (*n* = 2; 16.7%), and transient intraprocedural pain (*n* = 3; 25%). No patients reported dermal ulcers, peripheral paresthesia, or muscle weakness. Percentages were calculated per procedure (12 procedures on 10 patients).

## 4. Discussion

Our study demonstrates a varied response to ultrasound-guided injection therapy in patients with chronic non-cervical-origin interscapular pain. While half of the patients experienced substantial pain relief, the other half continued to report moderate to severe pain despite treatment. This variability is consistent with previous findings. For instance, Lee et al. observed sustained pain relief in 40% of patients receiving medial branch blocks and in 65% of those undergoing direct intra-articular facet joint injections after six months [9]. Similarly, Modi et al. documented a 40% clinical success rate at 12 weeks with the 5-in-1 injection technique [10]. Manchikanti et al. reported a higher success rate, with 76% of patients experiencing pain relief after medial branch blocks, although some required up to 8–12 repeated treatments over three years [18]. Radiofrequency ablation has also been widely used for cervical facetogenic pain, with reported pain relief rates of approximately 60% in selected patients [19]. However, both medial branch blocks and radiofrequency ablation primarily target cervical-origin pain, which was excluded from the present cohort. In contrast, our study focused on non-cervical-origin interscapular pain, for which established ablative techniques are less applicable. Taken together, these findings highlight the heterogeneous nature of chronic interscapular pain and underscore the limitations of currently available interventional pain management strategies. Within this feasibility framework, our goals were to confirm procedural practicality and short-term safety and to estimate effect sizes to guide the design of a definitive trial.

To address cases refractory to ultrasound-guided injection therapy, our study evaluated the efficacy of TAE for chronic, non-cervical-origin interscapular pain. Our results indicate that TAE provides significant and sustained pain relief for up to six months without severe adverse events. At six months post-treatment, the NRS score showed a reduction of approximately 70%, consistent with the findings of Shibuya et al., who first reported the effectiveness of TAE for trapezius myalgia, demonstrating significant pain reduction and functional improvement [16].

Notably, pain relief with TAE was comparable to that of the injection-only group at 1, 3, and 6 months, suggesting its potential as a salvage therapy for refractory cases. We observed that patients with higher baseline pain levels had a lower response rate to initial injections, ultimately requiring TAE. Although these differences were not statistically significant, a trend toward longer pain duration and a higher proportion of male patients was observed in the TAE group. These differences may reflect a more severe inflammatory condition, a distinct clinical phenotype with underlying pathophysiological mechanisms less responsive to injection therapy, or potential selection bias due to the small cohort size. Currently, limited literature exists on prognostic factors for interscapular pain in relation to specific invasive treatments. Further studies are needed to refine treatment selection and optimize clinical outcomes.

The analgesic effect of TAE is likely achieved by reducing pathological neovascularization and modulating inflammatory mediators involved in pain generation. This process may also alleviate vasodilation, increased vascular permeability, and chronic muscle inflammation associated with pain. Yoshida et al. reported that inflammation and angiogenesis primarily occur in the fascia rather than in the muscles in patients with dermatomyositis [20]. This concept inspires the importance of targeting the interfascial plane in interscapular pain treatment. This anatomical region, where the dorsal scapular nerve and spinal accessory nerve are commonly located, is also a key target of the 5-in-1 injection technique for relieving interfascial compartment pressure [10,21]. Compared to ultrasound-guided 5-in-1 injections, embolization with IPM/CS via the dorsal scapular artery (also known as the deep branch of the transverse cervical artery) may offer more extensive anti-inflammatory and analgesic effects across the entire arterial territory through a single vascular access, potentially enhancing treatment efficacy for refractory cases, particularly in patients with widespread or recurrent symptoms.

The safety profile of TAE for interscapular pain in this study was favorable, with no severe or life-threatening adverse events reported. Mild, self-limiting adverse events, including transient radial artery spasm, transient intraprocedural pain, cutaneous erythema, and puncture site hematoma, were observed but resolved without intervention. These findings align with previous studies on TAE for trapezius myalgia [16], reinforcing its reputation as a safe and well-tolerated procedure. Nevertheless, the relatively high frequency of minor adverse events merits closer scrutiny, particularly when evaluating the cost-benefit ratio of TAE in comparison to less invasive treatments such as physical therapy, oral medications, or ultrasound-guided injections. While TAE provides a unique therapeutic mechanism by targeting pathologic neovascularization and neurogenic inflammation, its invasiveness and catheter-based nature inherently carry procedural risks that must be justified by substantial clinical benefit. From a technical perspective, the transverse cervical artery—the primary target of this procedure—does not supply the spinal cord, thereby reducing the risk of non-target embolization. Additionally, prior research has demonstrated the selective embolization effect of IPM/CS, showing automatic recanalization in normal tissue while maintaining embolization in inflamed tissue, as observed in a rabbit model using microangiography [22]. This suggests a targeted effect on neovascularization, further supporting the procedure’s safety. However, despite the absence of severe adverse events in current studies, caution remains necessary due to the anatomical proximity of the transverse cervical artery—typically a branch of the thyrocervical trunk—to the vertebral and ascending cervical arteries, which have direct blood supply or anastomoses to the central nervous system. While the risk is low, the possibility of thromboembolic events or contrast reflux irritation cannot be entirely dismissed. Previous studies have reported transient adverse events, such as temporary hearing impairment and cortical blindness, following shoulder TAE [23,24]. Therefore, meticulous technique and careful procedural execution remain essential to minimize potential risks. 

Our study has several limitations. First, this work was intentionally structured as a retrospective feasibility study with a small cohort, aimed at establishing safety, procedural workflow, and an efficacy signal to inform future trial design. The limited sample size reduces statistical power and restricts generalizability. Second, the follow-up duration was only 6 months, which is insufficient to evaluate the durability of pain relief and long-term outcomes. Third, outcomes were assessed solely with the NRS for pain, without incorporating functional or quality-of-life measures, thereby limiting the comprehensiveness of the clinical evaluation. Fourth, the absence of randomization introduces a risk of selection bias. In particular, patients in the TAE group had significantly higher baseline pain levels than those in the injection-only group, which may have influenced the magnitude of observed pain reduction. Fifth, patients were not blinded to their treatment, and all outcomes were self-reported, raising the possibility of placebo effects and subjective bias. Finally, the selection of patients for TAE was based on refractory symptoms unresponsive to conservative therapies, whereas patients in the injection-only group generally experienced a clinical response. This discrepancy introduces confounding by indication, and any comparisons between the two groups should be interpreted with caution, as differences in baseline clinical status may have affected the outcomes independent of the intervention itself. Future prospective studies should include a larger cohort, longer follow-up, and randomization. They should also incorporate pain, functional, and quality-of-life measures to more definitively establish the efficacy of this treatment.

## 5. Conclusions

In this retrospective feasibility study, TAE appeared safe and showed potential for clinically meaningful, sustained pain reduction in patients with refractory non-cervical-origin interscapular pain after unsuccessful ultrasound-guided injections. Larger prospective—and ideally randomized—studies are warranted to validate efficacy, refine selection, and optimize outcomes.

## Figures and Tables

**Table 1:** Baseline patient characteristics.

Patient Characteristics	Injection-Only	Injection + TAE	*p* Value
Number	10	10	
Age, years	48.0 ± 10.1	50.1 ± 9.1	0.63
Sex (male/female)	2/8	7/3	0.07
Body Mass Index (kg/m^2^)	22.9 ± 2.5	22.6 ± 2.3	0.76
Pain duration (months)	6.9 ± 7.8	55.8 ± 73.3	0.07
Affected side, right/left/bilateral	2/6/2	5/5/0	—
Baseline NRS	5.6 ± 0.5	7.4 ± 1.4	0.003
Prior Conservative Treatment			
Oral NSAIDs	70% (7/10)	100% (10/10)	0.21
Oral Opioid	10% (1/10)	60% (6/10)	0.06
Rehabilitation	100% (10/10)	100% (10/10)	1.0
Type of injection therapy			
Intramuscular injection	90% (9/10)	100% (10/10)	1.0
Nerve Block/hydrodissection	10% (1/10)	50% (5/10)	0.14

Data are presented as means ± standard deviation or as number (percentage). TAE = transarterial embolization; NRS = numeric rating scale; NSAIDs = nonsteroidal anti-inflammatory drugs. *p* values were calculated using the independent *t*-test or Fisher’s exact test.

**Table 2:** Pain assessment post injection and transarterial embolization.

	TAE Group		Injection-Only Group		*p* Value (Score)	*p* Value (Percentage)
	Score Change	Percentage Change	Score Change	Percentage Change		
Post-Injection NRS						
Month 1	−2.2 ± 1.2	−28.7 ± 13.1%	−4.0 ± 0.7	−71.4 ± 9.2%	0.001	<0.001
Month 3	−1.4 ± 1.3	−18.2 ± 15.4%	−3.9 ± 0.7	−69.7 ± 11.4%	<0.001	<0.001
Post-TAE NRS						
Month 1	−5.3 ± 1.6	−70.2 ± 13.9%	—	—	0.024 *	0.931 *
Month 3	−5.4 ± 1.3	−73.4 ± 11.9%	—	—	0.005 *	0.486 *
Month 6	−5.5 ± 1.4	−74.8 ± 14.7%	—	—	0.006 *	0.397 *

Data are presented as means ± standard deviation. NRS = Numeric rating scale; TAE = Transarterial embolization. Score change = follow-up NRS minus baseline; percentage change = (follow-up − baseline)/baseline ×100% (negative = improvement). Data are mean ± SD. * Compared with injection-only group at Month 3.

**Table 3:** Use of conservative treatments for interscapular pain over time after transarterial embolization.

Treatment	Before TAE	1 Month	3 Months	6 Months
Oral NSAIDs				
Daily	10/10 (100%)	5/10 (50%)	2/10 (20%)	1/10 (10%)
As needed	10/10 (100%)	6/10 (60%)	2/10 (20%)	2/10 (20%)
Opioid				
Daily	7/10 (70%)	5/10 (50%)	2/10 (20%)	2/10 (20%)
As needed	8/10 (80%)	2/10 (20%)	1/10 (10%)	1/10 (10%)
Rehabilitation				
Daily	10/10 (100%)	5/10 (50%)	2/10 (20%)	2/10 (20%)
As needed	10/10 (100%)	0/10 (0%)	0/10 (0%)	0/10 (0%)

Data are *n*/*N* (%). “Daily” = routine daily use; “As needed” = intermittent use. TAE = transarterial embolization; NSAIDs = nonsteroidal anti-inflammatory drugs.

## Data Availability

The original contributions presented in this study are included in the article/Supplementary Material. Further inquiries can be directed to the corresponding authors.

## Supplementary Materials

The following supporting information can be downloaded at: https://www.mdpi.com/article/10.3390/diagnostics15192496/s1, Table S1: Individual patient-level data of the transarterial embolization group.
