# Supplementary material for: Transarterial Embolization for Refractory Non-Cervical-Origin Interscapular Pain Following Ultrasound-Guided Injection: A Retrospective Feasibility Study
Source: Diagnostics (Basel). 2025 Oct 1;15(19):2496. doi: 10.3390/diagnostics15192496 (PMC12523267; doi:10.3390/diagnostics15192496)
Supplement: Supplementary file 1 [file diagnostics-15-02496-s001.zip › TableS1_20250930.pdf]

Table S1. Individual patient-level data of the TAE group

Case No.	Sex / Age (years) / BMI (kg/m²)	Symptom Duration (M)	Type of Injection	TAE Target Artery	IPM/CS Dose (mg)	NRS prior Injection	NRS prior TAE	NRS post TAE 1M	NRS post TAE 3M	NRS post TAE 6M
1	Male / 52 / 21.2	36	S, D, PRP, NB	dTCA	*150	9	5	3	4	2
2	Male / 72 / 25.3	120	S, D, PRP	dTCA, sTCA	250	7	4	2	1	1
3	Female / 46 / 22.2	4	S, D, PRP, NB	dTCA, sTCA	250	8	7	1	1	1
4	Female / 56 / 25.1	6	S, D, PRP, NB	dTCA, sTCA, CSA	*200	8	6	2	3	2
5	Male / 52 / 18.2	8	S, D, NB	dTCA	150	8	7	2	2	3
6	Male / 50 / 24.1	24	S	dTCA	100	7	7	2	2	1
7	Male / 45 / 24.5	240	S, D, NB	dTCA	100	8	7	1	1	1
8	Male / 41 / 20.5	24	S, D, NB	dTCA	100	5	4	2	1	1
9	Male / 41 / 23.2	36	S	dTCA, sTCA, CSA	200	5	5	3	2	2
10	Female / 46 / 21.5	60	S, D, PRP, NB	dTCA	200	9	8	3	3	5

Abbreviations: No., number; BMI, body mass index; M, month(s); TAE, transarterial embolization; IPM/CS, imipenem/cilastatin sodium; NRS, numeric rating scale; S, intramuscular steroid; D, intramuscular dextrose; PRP, intramuscular platelet-rich plasma; NB, nerve block or hydrodissection; dTCA, deep branch of the transverse cervical artery; sTCA, superficial branch of the transverse cervical artery; CSA, circumflex scapular artery.

* Indicates that two TAE sessions in the same patient were performed with the same IPM/CS dose.
